# Supplementary material for: Evaluation of Firefly and Renilla Luciferase Inhibition in Reporter-Gene Assays: A Case of Isoflavonoids
Source: Int J Mol Sci. 2021 Jun 28;22(13):6927. doi: 10.3390/ijms22136927 (PMC8268740; doi:10.3390/ijms22136927)
Supplement: Supplementary file 1 [file ijms-22-06927-s001.zip › ijms-1257379-supplementary.pdf]

Evaluation of Firefly and *Renilla* Luciferase Inhibition in Reporter-Gene Assays: A Case of Isoflavonoids

Supplementary Materials

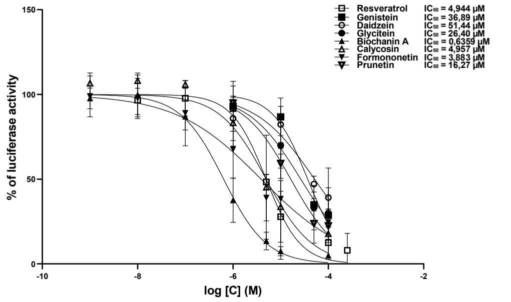

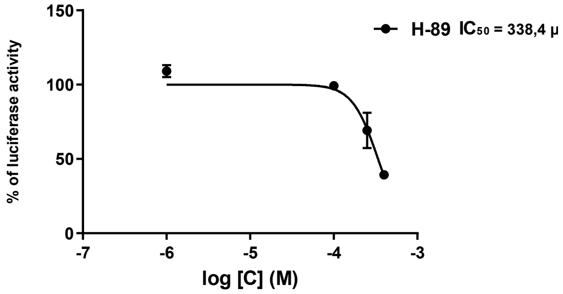

(**a**) (**b**)

Figure S1. Dose-response curves for firefly luciferase (**a**) and *Renilla* luciferase (**b**) inhibition. (**a**) Results of the *in-vitro* firefly luciferase inhibition assays for the positive vcontrol, resveratrol, and the isoflavonoids. (**b**) Results of the *in-vitro Renilla* luciferase inhibition assay for the positive control. H-89. All of the data were normalized to the relevant controls (1% dimethylsulfoxide). Data are means ±standard deviation of three independent repeats in triplicates.

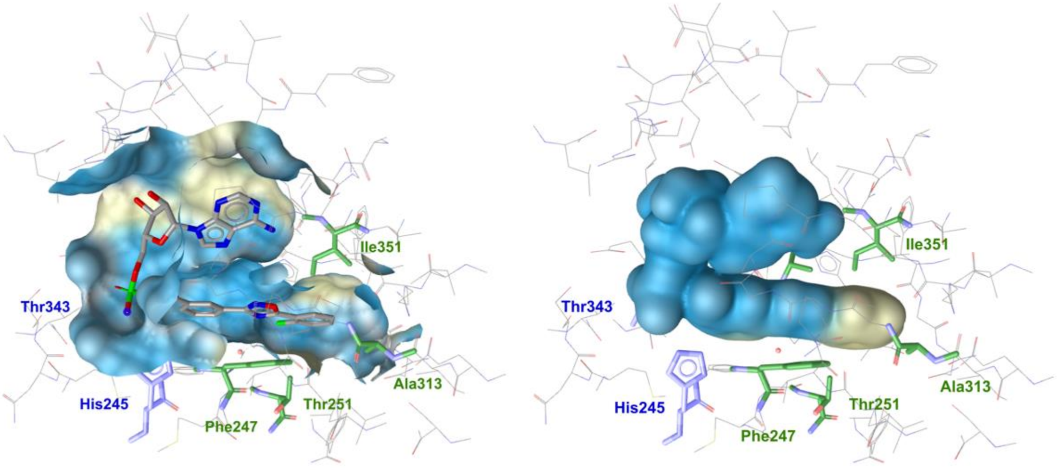

(**a**) (**b**)

Figure S2. Firefly luciferase. (**a**) Three-dimensional representation of the binding site with bound PTC124-AMP ligand. (**b**) Molecular surface representation of the bound PTC124-AMP ligand volume (PDB:3IES).

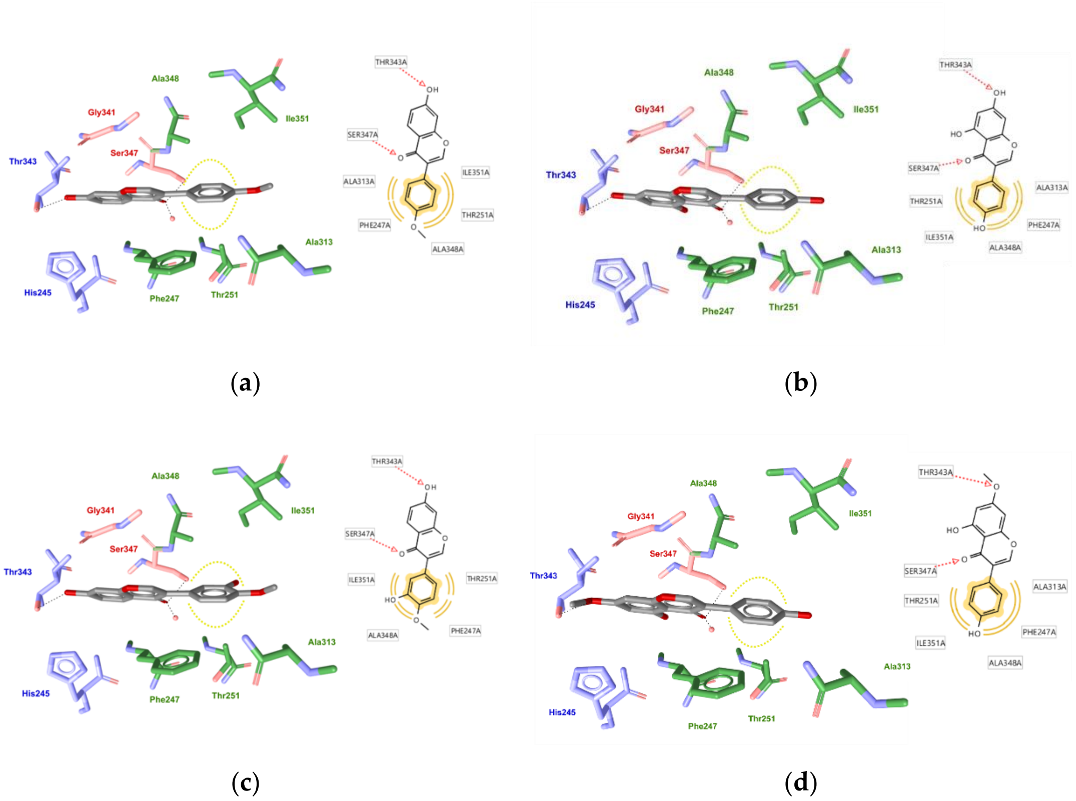

Figure S3. Firefly luciferase active site (PDB:3IES). Representative GOLD binding modes (left) and crucial interactions identified (as two-dimensional schemes; right) for the active isoflavonoids formononetin (**a**), genistein (**b**), calycosin (**c**) and prunetin (**d**).

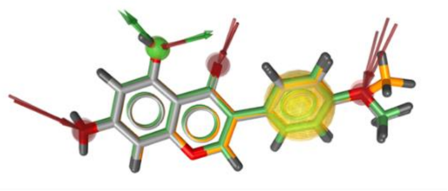

(**a**)

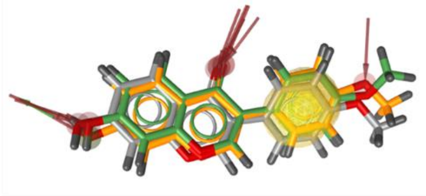

(**b**)

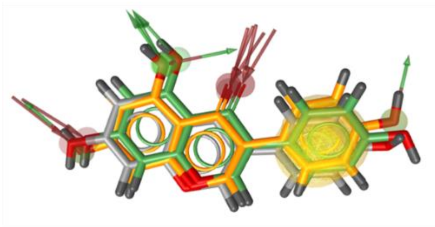

(**c**)

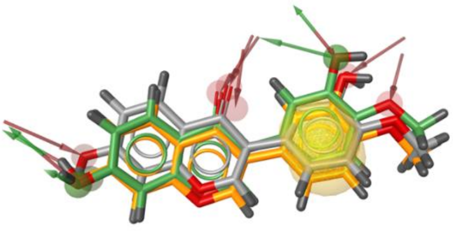

(**d**)

Figure S4. Comparisons of GOLD binding modes and interactions identified in LigandScout for the active isoflavonoids biochanin A (**a**), formononetin (**b**), genistein (**c**), and calycosin (**d**), when docked in the 3IES, 3RIX 4E5D firefly luciferase structures, with protein structures aligned (protein environment not shown).

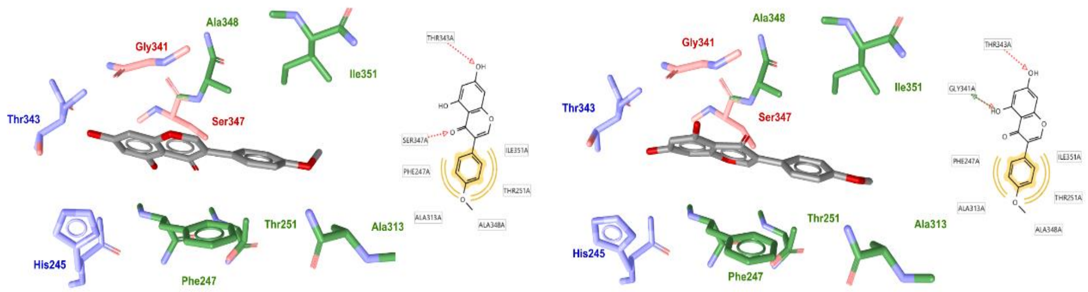

Figure S5. Firefly luciferase active site (PDB:3IES). Two orientations of biochanin A GOLD binding modes using water-free binding site, and two-dimensional schemes of important intermolecular interactions (right).

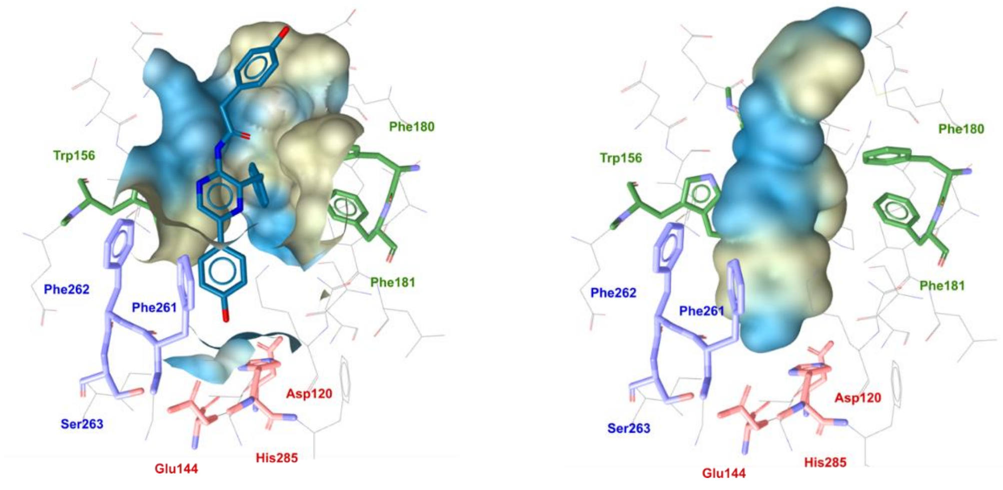

(**a**) (**b**)

Figure S6. *Renilla* luciferase. (**a**) Three-dimensional representation of the binding site with bound ligand coelenteramide. (**b**) Molecular surface representation of the bound ligand coelenteramide volume (PDB:2PSJ).

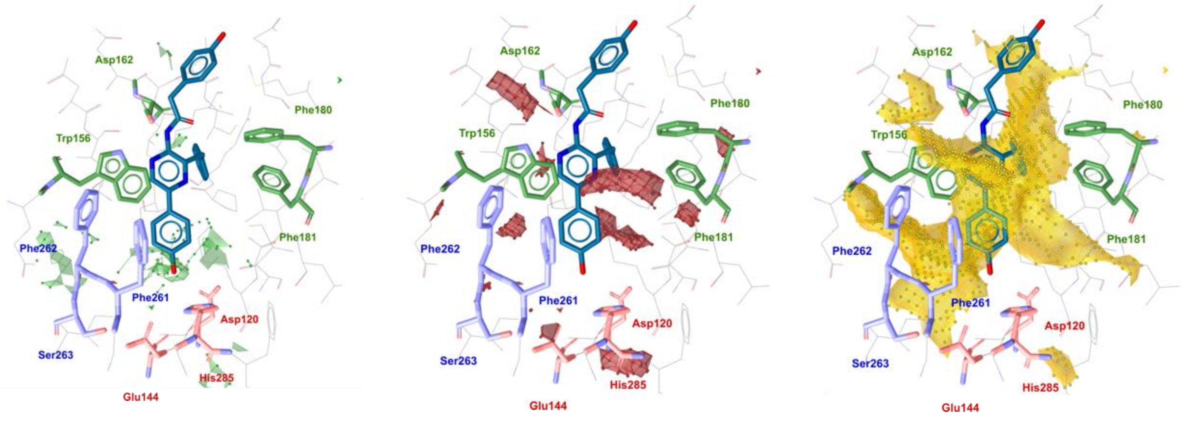

(**a**) (**b**) (**c**)

Figure S7. *Renilla* luciferase active site. Calculated molecular interaction fields using hydrogen bond donor (**a**; green), hydrogen bond acceptor (**b**; red) and hydrophobic (**c**; yellow) probes, with the bound ligand coelenteramide shown (PBD: 2PSJ).

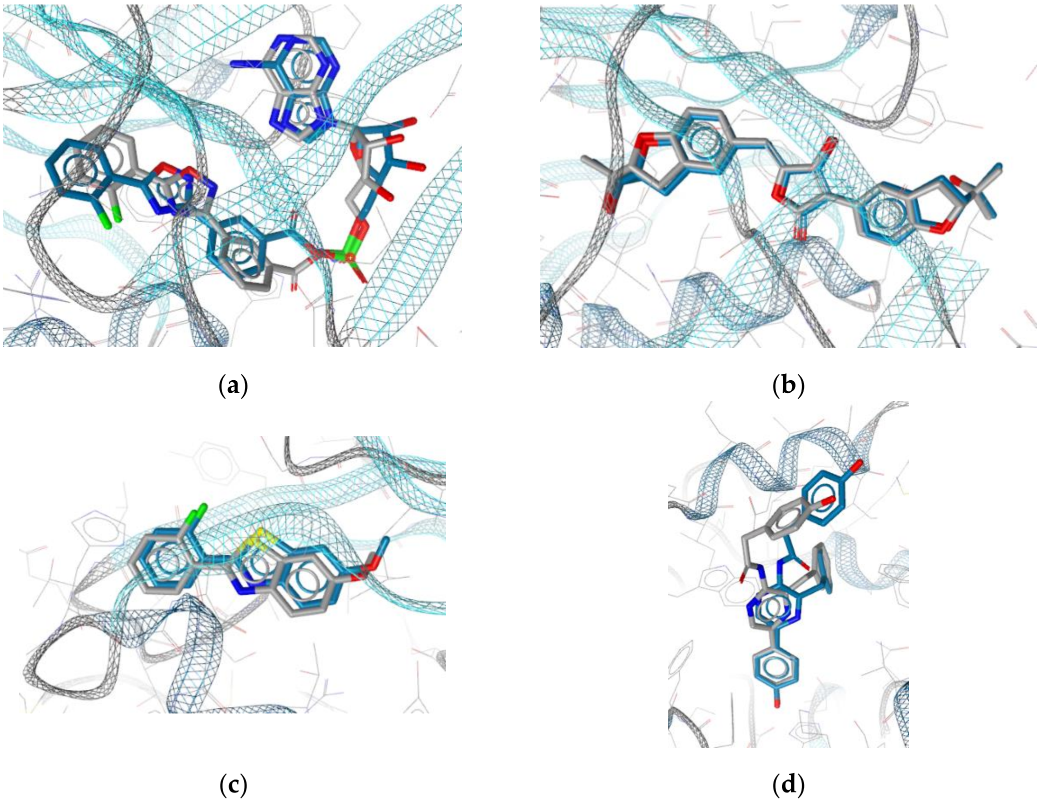

Figure S8. Comparisons of the docked and X-ray conformations of various ligands in the firefly luciferase (**a-c**) and *Renilla* luciferase (**d**) binding sites. (**a**) PDB:3IES: RMSD, 0.75 Å between heavy atoms of PTC124-AMP poses. (**b**) PDB:3RIX: RMSD, 0.42 Å between heavy atoms of aspulvinone J-CR poses. (**c**) PDB:4E5D: RMSD, 0.75 Å between heavy atoms of benzothiazole poses. (**d**) PDB:2PSJ: RMSD, 1.41 Å between heavy atoms of coelenteramide poses.
